# Thermodynamically induced *in Situ* and Tunable Cu Plasmonic Behaviour

**DOI:** 10.1038/s41598-018-20478-y

**Published:** 2018-02-14

**Authors:** Gajendra Kumar Inwati, Yashvant Rao, Man Singh

**Affiliations:** 1 Centre for Nanosciences, Central University of Gujarat, Gandhainagar, 382030 India; 2 School of Chemical Sciences, Central University of Gujarat, Gandhainagar, 382030 India

## Abstract

The Cu nanoparticles (Cu NPs) were grown in soda-lime glass matrix through Cu^+^ ↔ Na^+^ ion exchange methods under thermal annealing in an open environment and studied variation in their size on tunable plasmonic behaviour, optical absorption spectra and photoluminescence (PL). A blue shift from 570 to 560 nm was observed in localized surface plasmon resonance (SPR) of Cu NPs from 550 to 650 °C. A mutual relation between size and surface plasmon resonance with full width half maxima (FWHM) has been derived for plasmonic properties at variable temperatures. Structural investigations of embedded Cu NPs have been confirmed by using HRTEM and EDX. Grazing incidence X-ray diffraction (GIXRD) had identified a crystalline nature of Cu NPs under annealed conditions. XPS, Raman and secondary ion mass spectroscopies (SIMS) have identified an embedding behaviour of Cu NPs in glass matrix. Plasmonic and thermodynamic properties of embedded Cu NPs have explained their *in situ* thermal growth mechanism for efficient distribution where enthalpy (∆H), entropy (∆S) and Gibbs free energy (∆G) have interpreted their temperature driven Cu NPs growth. An interdependence of ∆H, ∆S and ∆G has been developed vis-a-vis activation energy on an extent of 12.54 J/mol.

## Introduction

For last few decades, the field of plasmonics has become a thrust area of research for developing the concepts and applications through the interactions of electromagnetic with free electrons of metal nanostructures^1^. Plasmon-based Cu, Ag and Au noble metallic structures have gained tremendous scientific interest due to their tunable plasmonic and catalytic properties at nanoscale for not only efficient process but also a quality of nanomaterials^2–4^. Their nanostructures are substantially used in fields of optoelectronics, nanophotonics, biotechnology and others due to their unique and unusual physicochemical and functionalities^5–9^. In plasmonic metals, nanosize Cu clusters embedded in dielectric matrix have attracted great attention because of their tunable longitudinal SPR activities due to collective motions of conduction band electrons when are induced by electromagnetic radiations^10,11^. Such optically sensitive localized resonance frequency significantly used for photovoltaic applications when it falls in a visible or infrared region. The coupled electromagnetic frequency of metallic NPs could open a new insight for advanced cancer cell treatment, biomolecule sensing and solar cell applications. Thereby, the resonance frequency of metallic NPs could be tuned by selecting their compositions, dielectric medium, and interparticle distances of dispersed nanoparticles^12,13^. In particular, a transparent silicate glass embedded with nanoscopic Cu metal is the best choice because of its ultra large third orders nonlinear susceptibility and ultrafast effect^14,15^. Therefore, soda-lime glass matrices have been used as a host material due to its mechanical strength, higher transparency and easy processing to grow Cu NPs. As per our intense literature survey, the limited studies are reported to materialize such concepts and study gaps create a need to enhance the database of such materials with several metals in varieties of matrices. Recently, many approaches are addressed to grow the metallic nanoparticles inside the host matrix like ion implantation, melt-quench techniques, low energy ion-beam mixing, and physical vapour deposition for targeted and specified functions^16,17^. A critical review of the reported methods used for synthesis had revealed few demerits of using larger amounts and duration but an ion-exchange method which we have adopted needs a less concentration with shorter ion-exchange duration. The arrays of metallic NPs could be oriented under thermal or irradiation treatment for producing a fine size distribution of nanosize particles inside the soda-lime glass matrix. Thereby, the thermal annealing was preferred with a higher precision and better control over a particle size, which is advantageous over other methods used for the purpose.

Further, we have proposed a temperature induced dissociation, ionization, and redox reactions systematically for growing Cu NPs on a soda-lime glassy matrix mixing 0.5% of CuSO_4_ with 95.5% of Na_2_SO_4_. The Cu ion exchange has been made at 580 °C within a short a duration as compared to the reported work^11,18^. The chosen ratio of CuSO_4_ and Na_2_SO_4_ has adequately reduced the Cu^+^ to Cu^0^ under thermal conditions. The Cu particles are structurally oriented as NPs under uniform annealing up to 650 °C for 1 h inside the dielectric matrix. The objectives of our work have been to study the tunable plasmonic behaviour with thermodynamics of Cu-doped soda-lime glass from 550 to 560 °C. The structural, optical, and thermodynamic properties of as-grown Cu NPs have been investigated and explained with a mutual relevance. Moreover, the thermodynamics has revealed the significant information about the physicochemistry by considering ∆H, ∆S and ∆G^16,19^. Hence an annealing temperature and time both play a key role for growing a material under controlled morphology with surrounded medium where both the chemical and physical interactions of components regulate the nucleation and particle growth with lattice orientations by mass and heat-transfer activities^20,21^.

## Results and Discussion

### UV-Vis spectroscopy analysis

**Figure 1:**
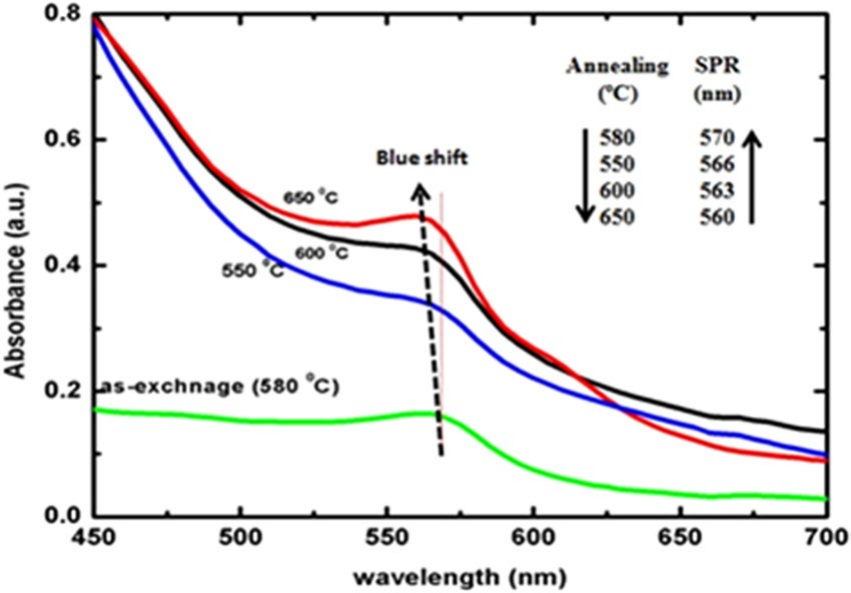
UV-Vis spectra of ion-exchanged and annealed Cu embedded glass materials.

**Figure 2:**
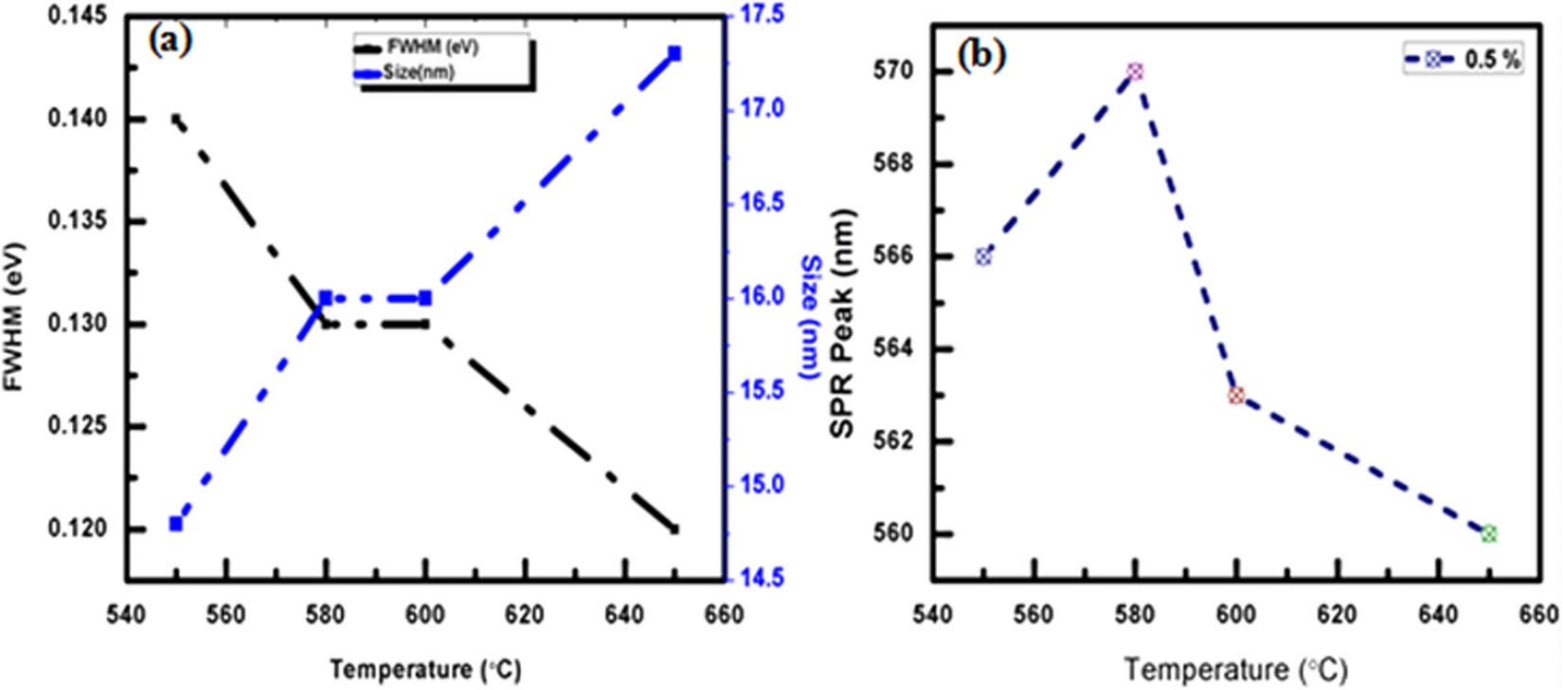
(**a**) FWHM and size w.r.t. temperature. (**b**) Annealing temperature w.r.t. SPR peak intensity.

Figure 1 shows UV-Visible absorption spectra for pristine and thermally annealed Cu-doped glass samples from 550 to 650 °C for 1 h. The pure glass slide was used as a reference during UV-Vis spectra measurements which nullified the substrate properties. A broader (low intensified) SPR peak is occurred at 570 nm and inferred that the few Cu NPs were formed of <1 nm after a 5 min ion-exchange at 580 °C (Fig. 1)^22,23^. The Cu particles exist in Cu^2+^ and Cu^+^ states inside a glass matrix where the Cu^+^ does not respond to visible spectra for UV-Vis measurement while the Cu^2+^ shows an optical response^24^. After an annealing operation, an absorption band was observed at 566–660 nm w.r.t. 550, 600 and 650 °C due LSPR band of Cu NPs inside the glass matrix. As a result, a blue shift of 10 nm of the LSPR band from 560–570 nm is observed with on increasing particle sizes, which lowers the FWHM values from 550–650 °C (Fig. 2a,b). In the optical absorption spectra, LSPR peak intensity is increased on increasing annealing temperature which is attributed to a temperature induced Cu NPs growth inside a glass matrix as Cu^2+^/Cu^+^ → Cu^0^. In this process, the glass acted as a host material and had provided required electrons to reduce the Cu ions into neutral Cu atoms. Therefore a temperature driven mechanism was developed for a reduction process and the source of electrons are discussed in mechanism. At a higher temperature, the electrons are captured from silicate species of the glass to reduce the Cu^+^ to Cu^0^. Therefore, the more Cu^+^ ions were reduced into neutral Cu^0^ atoms which infer higher volume fraction of Cu NPs in glass matrix^25,26^. The size, SPR and FWHM are calculated for pristine and annealed samples to investigate thermal effect on size of Cu NPs, values are given in Table 1.

**Table 1:** SPR, FWHM, size values at temperatures from 550 to 650 °C at 1 h.

Annealing Temperature (^о^C)	Annealing Time (h)	SPR (nm)	FWHM (eV)	Size (nm)
550	1	566	0.14	14.5
580	1	570	0.13	16.5
600	1	563	0.13	16.5
650	1	560	0.12	17.3

In Table 1, on increasing temperature, Cu NPs increase in size which infers a larger partitioning to favour a more clusterification that is why the FWHM energy change is slightly decreased on increasing Cu NPs size. Thus, the Cu NPs homogenise at 650 °C where the FWHM energy is counterbalanced rather than align for electrostatic lining. Thus a decrease in FWHM supports comparatively a larger distribution at higher temperature in definite arrays. So on increasing the temperature the SPR decreases at the rate of 10.24, −17.95 and −40.86 nm/Kelvin because of larger partition of Cu NPs under annealing. The FWHM remained constant from 580 to 600 °C and within this range the size also remained constant (Table 1). For plasmonic behaviour of Cu embedded NPs, the size-derived permittivity ε (*ω*, *R*) of the Cu NPs and absorption extinction (K) of Cu NPs affect the processes. The R ≪*λ, and λ* is wavelength of light under quasi-static or dipole-dipole approximation^27^:

1 $$\varepsilon (\omega ,r)={\varepsilon }_{1}(\omega )+{\mathfrak{i}}\,{\varepsilon }_{2}(\omega ,\,r)$$

The resonance occurs when the *ε*_1_(*ω*) = −2 *ε*_*m*_ is fulfilled and in case of SPR, the light field induces a resonant coherent oscillation of free electrons across metal NPs. Optical coefficient (α) for Cu NPs surrounded by dielectric medium is expressed as^27^ –

2 $${\rm{\alpha }}=9\frac{\omega }{c}{\varepsilon }_{m}^{3/2}V\frac{{\varepsilon }_{2}(\omega )}{{[{\varepsilon }_{1}(\omega )+2{\varepsilon }_{m}]}^{2}}\frac{1}{+{\varepsilon }_{2}{(\omega )}^{2}}$$

where, ε_*m*_ dielectric constant of medium and V is volume fraction of metal particles which is smaller as compared to an imposed light wavelength (λ) and the ε_1_ and ε_2_ are frequency dependent real and imaginary components which are expressed by optical constant of their bulk metal^28^.

Average size of Cu NPs is calculated with equation-3where d = average size of particle,  Planck’s constant, V_f_ = Fermi velocity of electron of bulk Cu (1.57 × 10^6^ m/s), ∆E_1/2_ is FWHM of SPR band. The data are in a close agreement for a size of metal NPs which is smaller than a free mean path of electron which is 27 nm at RT for the bulk^29^. The average particle sizes were calculated by UV-Vis absorption spectra are 14.5, 16.5, 16.5 and 17.3 nm for 550, 580, 600 and 650 °C respectively (Fig. 2a).

### Photoluminescence spectra

**Figure 3:**
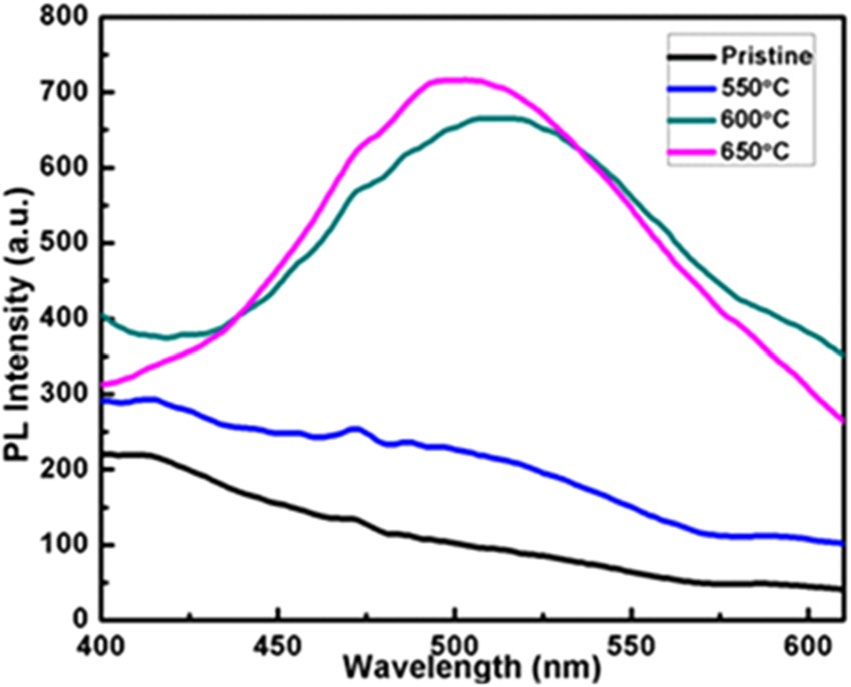
PL spectra of Cu ion-exchanged glass and annealed samples at various temperatures.

**Figure 4:**
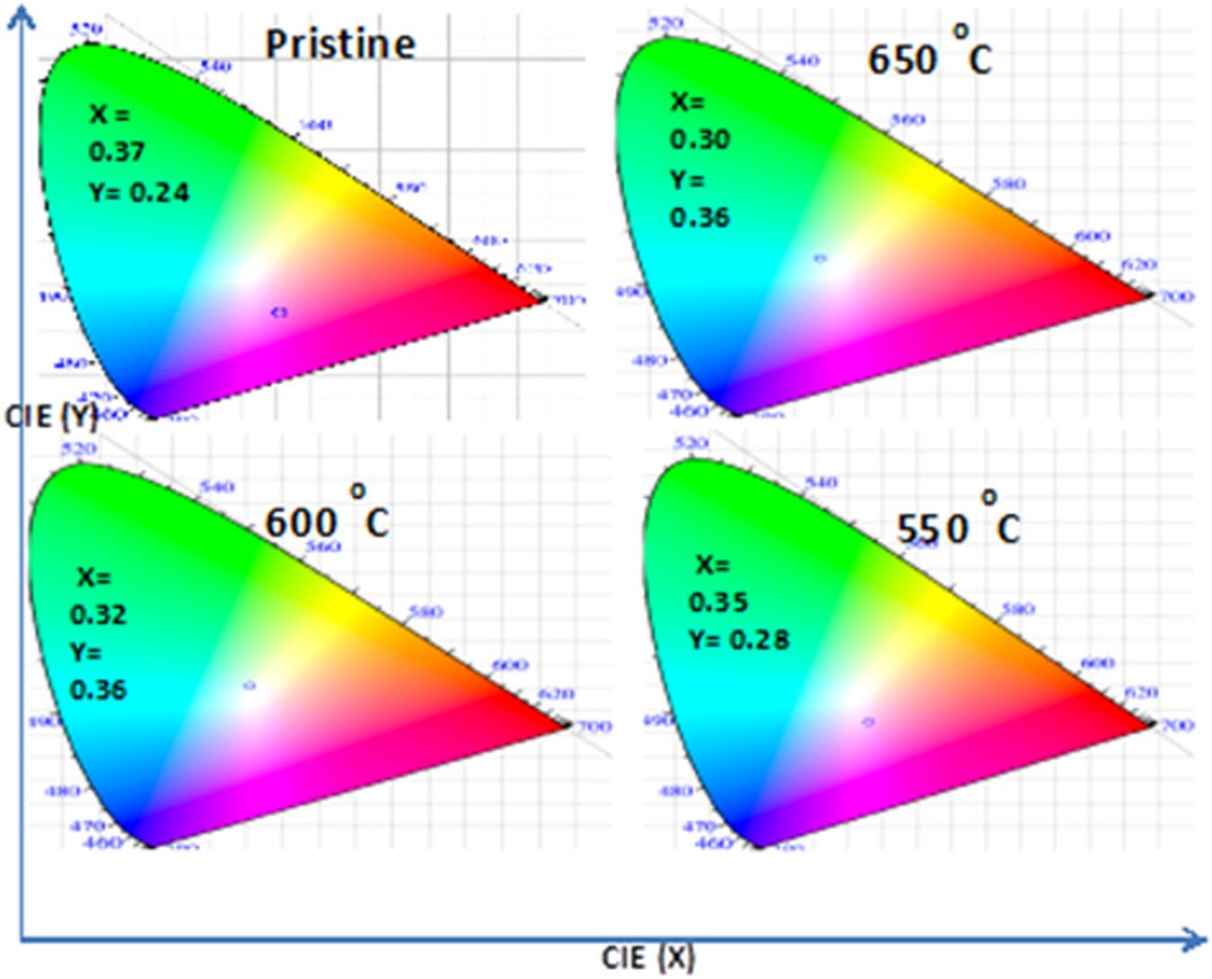
CIE diagram for pristine and annealed samples at various temperatures.

Figure 3 shows PL spectra of ion-exchanged (Cu^+^-Na^+^) and annealed samples from 550 to 650 °C for 1 h. It depicts that the larger numbers of the Cu NPs get activated with a higher energy that had caused a blue shift at 497 nm at 650 °C contrary to the 550 nm at 600 °C under the selected visible ranges (400–600 nm). Also the PL given in Fig. 4 infers the similar trends of spectroscopic energy holding ability of Cu NPs at higher temperature which distinguishes a role of kinetic energy at variable λ_max_. PL emission depends on a nature of glass matrix^30^ as a dielectric medium which facilitates Cu ions embedding inside dielectric materials. Similar to SPR and Cu NPs sizes (Table 1) and activation of larger numbers of Cu NPs with higher energy, the results of PL support a working mechanism of Cu NPs at higher temperature. Luminescence properties are analyzed for pristine and samples at 325 nm excitation wavelength and the CIE (Commission International de I’Eclairage) chromaticity plot shown in Fig. 4. The CIE identifies the colours by a luminance parameter and colour coordination x and y direction to specify the point at chromaticity diagram. The CIE diagram focuses an opacity order like blue or red shifts by measuring colour based spectral power distribution (SPD) of emitted light from sample. Correlated Colour Temperature (CCT) to x, y coordinates is integrated over a product of CIE 1931 2 degree λ functions and Planck’s law energy distribution. A use of CIE software facilitates a matching of blue shifts effect with the UV-Vis data (Fig. 1). PL data were fitted by CIE and plotted which give a clear sign about blue shift from pristine to annealed samples in their optical behaviour based on nature, alignment and size of cu NPs. Emission spectra of Cu embedded NPs were converted to CIE chromaticity using the PL data, and pristine sample tends towards a yellow region but the Cu NPs shows a blue shift which is also exhibited in UV-Vis spectra. CIE data for embedded Cu nanostructures infer multicolour emissions at 325 nm in visible zone at a single wavelength light. Thereby the optically sensitive properties could also be used in fields of optoelectronic, bioimaging, and light emitting diodes applications. The CIE diagrams through the changes in optical intensities have reflected a variation in size from 550–650 °C.

### GIXRD profile of Cu embedded soda-lime glass2

**Figure 5:**
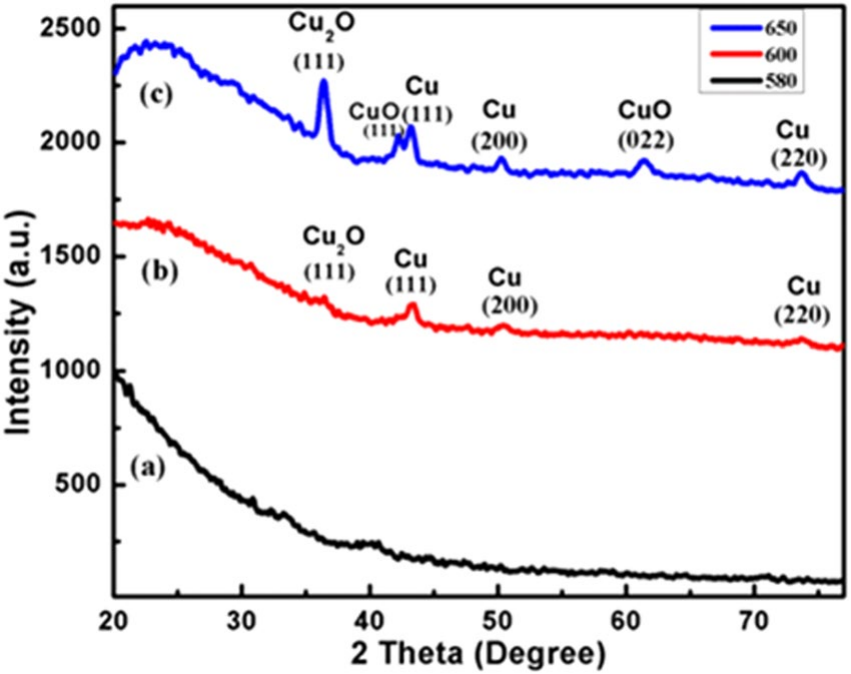
GIXRD pattern of Cu embedded glass samples; (**a**) pristine; (**b**) 600 °C; and (**c**) 650 ^о^C.

Figure 5 shows the GIXRD graph of as exchanged (pristine) and annealed sample at 600 and 650 °C where the GIXRD analysis enabled to understand the temperature induced effects on Cu nanocrystal formation inside glass matrix. Figure 5a illustrates the GIXRD pattern for pristine sample which indicate weakly intense peaks corresponding to Cu^0^ (002), Cu_2_O (111), Cu^0^ (022) and Cu^0^ (220) located at 35.7, 38.8, 66.2 and 68.4° 2*θ* positions respectively^31^. Pristine sample does not show any signals for pure Cu crystals except the baglike amorphous peaks. This may be due to a situation where no Cu crystals could have been formed during an ion-exchange or the crystals which had been formed in a very small size causing a broad diffraction peaks. The thermally annealed sample exhibited the Cu_2_O (111), Cu (111), Cu (200), and Cu (220) phase structures at 36.5, 43.2, 55.8, and 74.2° 2*θ* positions of respectively (Fig. 5b,c)^10,31^. The diffraction planes with (111), (200) and (220) closely agree with the standard card JCPDS No.04-0836, which had confirmed the Cu nanocrystals formation. Here the sharpened peak shows a better crystallinity of the grown Cu nanocrystals inside glass matrix. Thus the oxide (Cu_2_O/Cu^0^) species occurred due to bonding with intrinsic oxygen of SiO_2_ matrix under an open environment. But the annealed samples at 600 °C and 650 °C show the higher intense peaks for pure Cu NPs which indicate the higher population of Cu atoms under higher annealed temperature. The GIXRD results are correlated with XPS data which support a Cu NPs growth in the glass matrix at higher temperature.

### Raman spectra analysis

**Figure 6:**
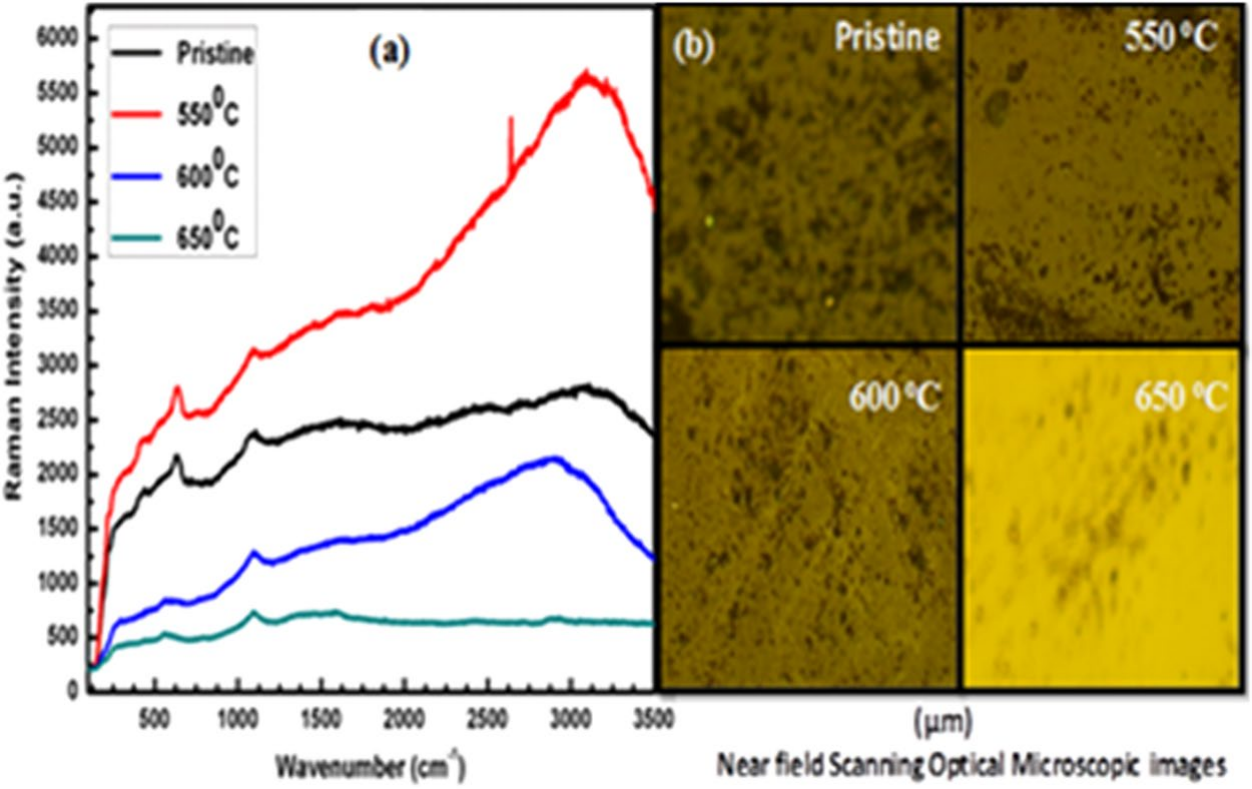
Raman spectra of (**a**) Cu pristine and annealed samples. (**b**) Microscopic images of Cu embedded glass surface.

Figure 6 shows Raman spectra of pristine and annealed Cu NPs samples from 550 to 650 °C where a green laser with 532 nm excitation wavelength was used for Raman analysis. Raman peak located at 625 cm^−1^ infers Cu NPs formation. The Raman spectra attribute Cu diffusion in soda-lime glass matrix on a thermally annealed condition. The Raman microscopic images are shown for pristine and annealed samples in Fig. 6b. In the Raman spectra, the pristine peak shows a higher intensity as the larger concentration of Cu ions occurs on glassy surface at 580 °C. In case of an annealed Cu samples, Raman spectra show a lower intense peak sequentially which may be due to a diffusion of Cu atoms in the glass matrix under higher temperature (Fig. 6a). These results indicate an exchange of larger number of Cu ions and embedded in the glass under temperature driven process. Raman spectra have supported larger Cu atoms diffusion into glass matrix with a higher kinetic rate on increasing temperature. A larger difference in peak intensity is appeared between pristine and Cu samples at 600 and 650 °C on account of Cu atoms diffusion following variable particles size distributions in the glass matrix. Surface accumulated Cu atoms are diffused towards relax stress (due to a difference between ionic radii of Cu^+^/Cu^2+^ and Na^+^) and balance a minimum energy^32^. The Cu^2+^ ions show a strong peak at 495 cm^−1^ which is associated with a shoulder at 575 cm^−1^, while a signature of the host matrix is appeared at ≈1015 cm^−1^. The peak ≈1000–1100 cm^−1^ is assigned to Si-O stretching vibration of silicate chains of the glass content^32,33^. The peaks at 580–630 and 1080 cm^−1^ indicate to the Cu^0^ embedded while the Cu^2+^ has produced the peaks at 490 and 1015 cm^−1^. This study helps to explain the embedding activities of Cu inside glass matrix under influence of temperature as the intensity changes sequentially in Raman spectra.

### Structural and crystallinity confirmation

**Figure 7:**
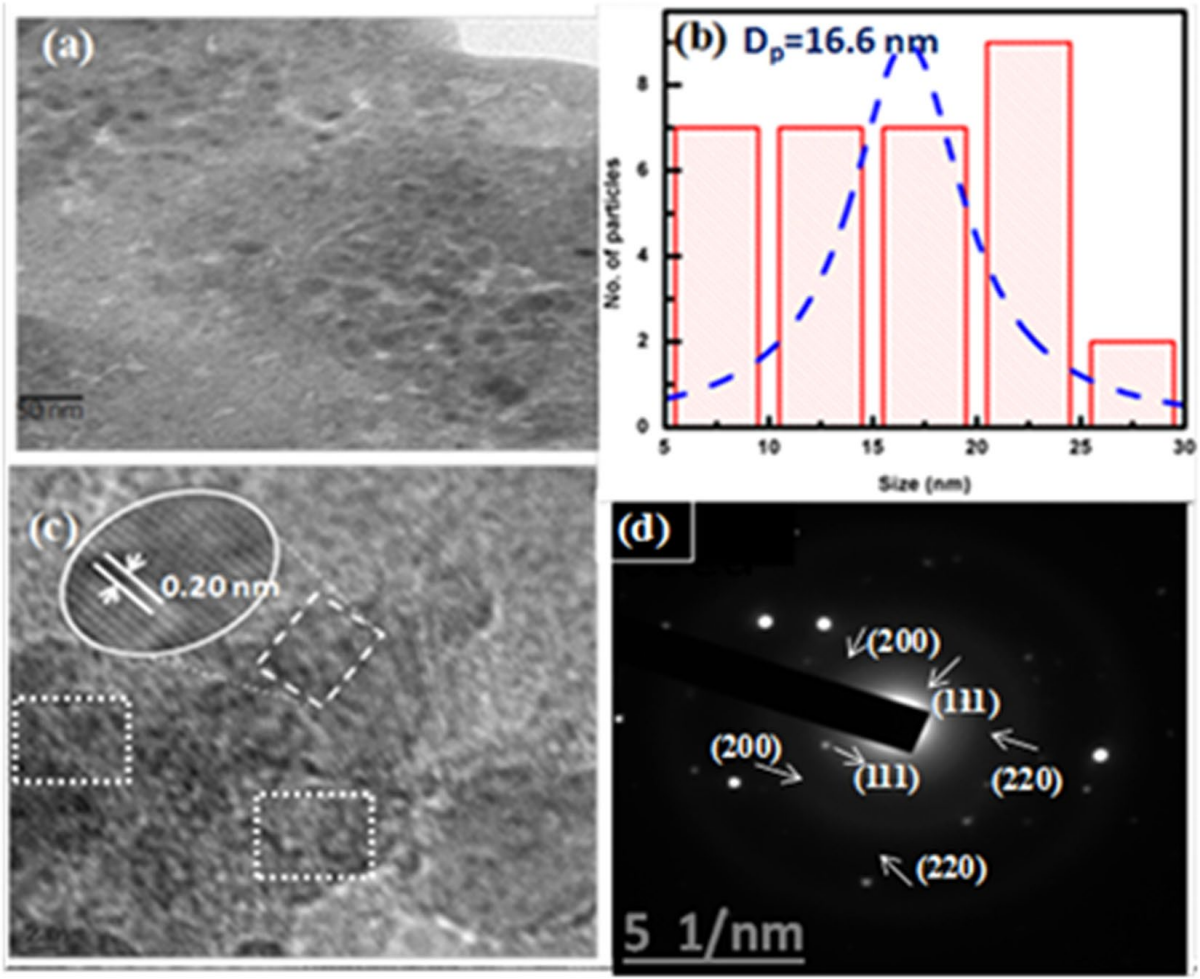
HRTEM image: (**a**) TEM image of Cu NPs at 650 °C. (**b**) Particle size distribution curve. (**c**) High resolution TEM images of Cu NPs. (**d**) SAED patterns.

**Figure 8:**
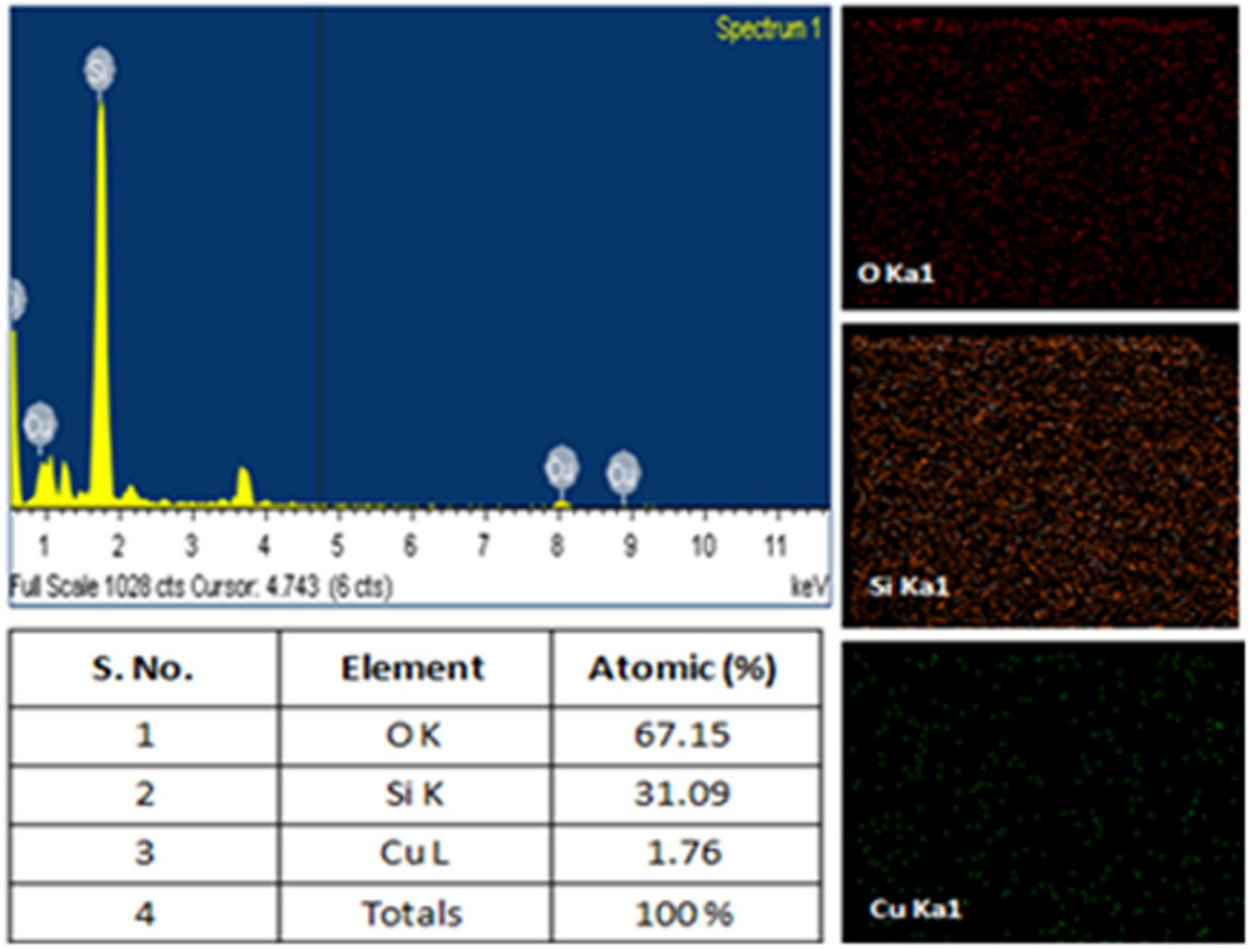
EDX spectra of Cu embedded glass sample annealed at 650 °C.

Transmission electron micrographs were taken to investigate the shape and size of embedded Cu NPs at higher temperature. Figure 7a shows spherical shape of Cu NPs at 650 °C with an average size of 16.6 nm (Fig. 7b). A higher resolution TEM image and selected area electron diffraction (SAED) patterns were fitted with the crystallographic planes and crystalline nature of Cu NPs at 650 °C. The spherical Cu NPs of (111) and (200), (220) crystallographic planes are observed in diffraction patterns (Fig. 7d). The standard of crystal lattice with 2.09 Å d-spacing (PDF no. 04-0836, 0.209 nm) shows an agreement of (111) plane of Cu NPs^31,34^ (Fig. 7c). The obtained crystallographic planes are correlated with GIXRD results which support Cu NPs growth at higher temperature. An average calculated Cu NPs size of 16.5 nm is a close agreement with UV-Vis spectroscopy (14.5, 16.5, 16.5 and 17.3 nm for 550, 580, 600 and 650 °C respectively) results. Figure 8 shows an elemental composition of Cu with some other extra peaks of Si and O which were sourced from glass matrix. The image mapping explains a presence of individual elements as the constituents. EDX spectra show an atomic percentage of elements with their K, L series respectively where a presence of Cu atoms is 1.76 atomic %.

### XPS Analysis

**Figure 9:**
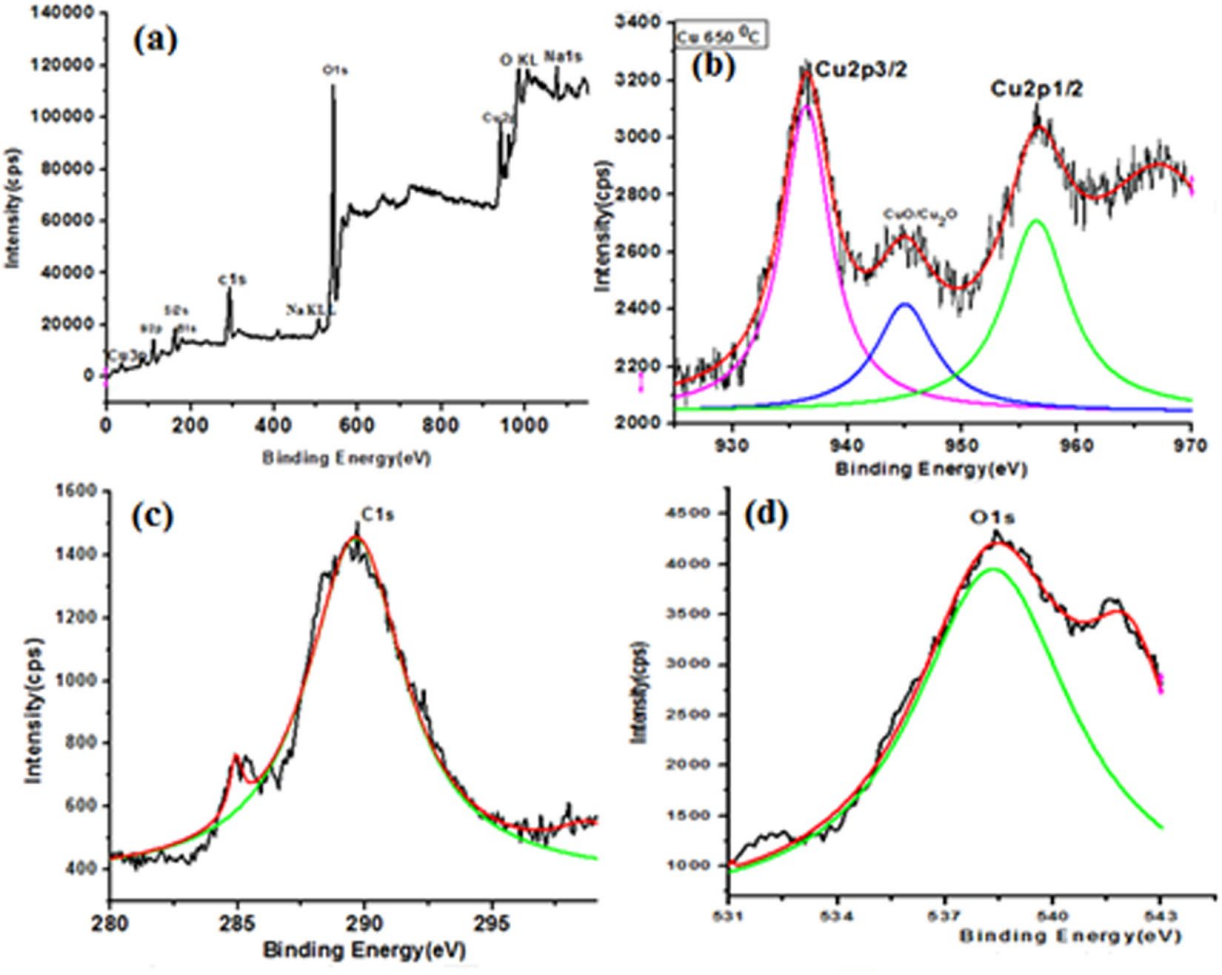
XPS spectra of Cu nanoparticle embedded glass: (**a**) XPS full spectrum and (**b**) Cu 2p high-resolution XPS spectrum (**c**) C 1s spectrum and (**d**) O 1s XPS peak.

XPS spectra identify an oxidation state of Cu embedded glass at 650 °C (Fig. 9). XPS measurements were taken using Omicron nanotechnology model with monochromatic Mg-Kα radiations (1253.6 eV) generated applying 15 KV electron impact on Mg anode. The pass energy was fixed at 20 eV to show a batter resolution of 0.5 eV. In the measurement, the Cu binding energy was calibrated with respect to the binding energy of C 1 s (284.6 eV). Figure 9a shows XPS full survey scan for the Cu embedded glass where the glass component appears with Cu signals. Figure 9b shows a high resolution X-ray photoelectron spectrum of Cu 2p for Cu diffused glass matrix with two peaks which correspond to Cu 2p3/2 and Cu 2p1/2. The 936.67 eV (2p3/2) and 956.65 (2p1/2), binding energy are obtained for Cu NPs^35^. The zero oxidation state binding energy is located at 936.67 eV (2p3/2) and 956.55 eV (2p1/2) for Cu in the spectra^36^. One more extra weak peak is appeared at 941.91 between two strong peaks may be due to a presence of Cu_2_O or Cu^0^ phase in glass matrix^37^. The sample may also have the Cu^2+^ state along with Cu neutral atoms which shows a miner peak in between Cu 2p3/2 and Cu 2p1/2 two major peaks. The data closely match with GIXRD for the Cu NPs formation with the presence of oxide species at higher temperature. The C1s and O1s as extra elements are appeared (Fig. 9c,d) because of the surrounding medium of the matrix or could be due to an environment of vacuum chamber of the instrument. These results are supported by SIMS data which show a presence of Cu_2_O and Cu^0^ both with a weakly intensified peak as compared to higher intense Cu NPs. The XPS and SIMS both results have confirmed a presence of neutral Cu atoms inside glass matrix.

### Atomic Force Microscopy

**Figure 10:**
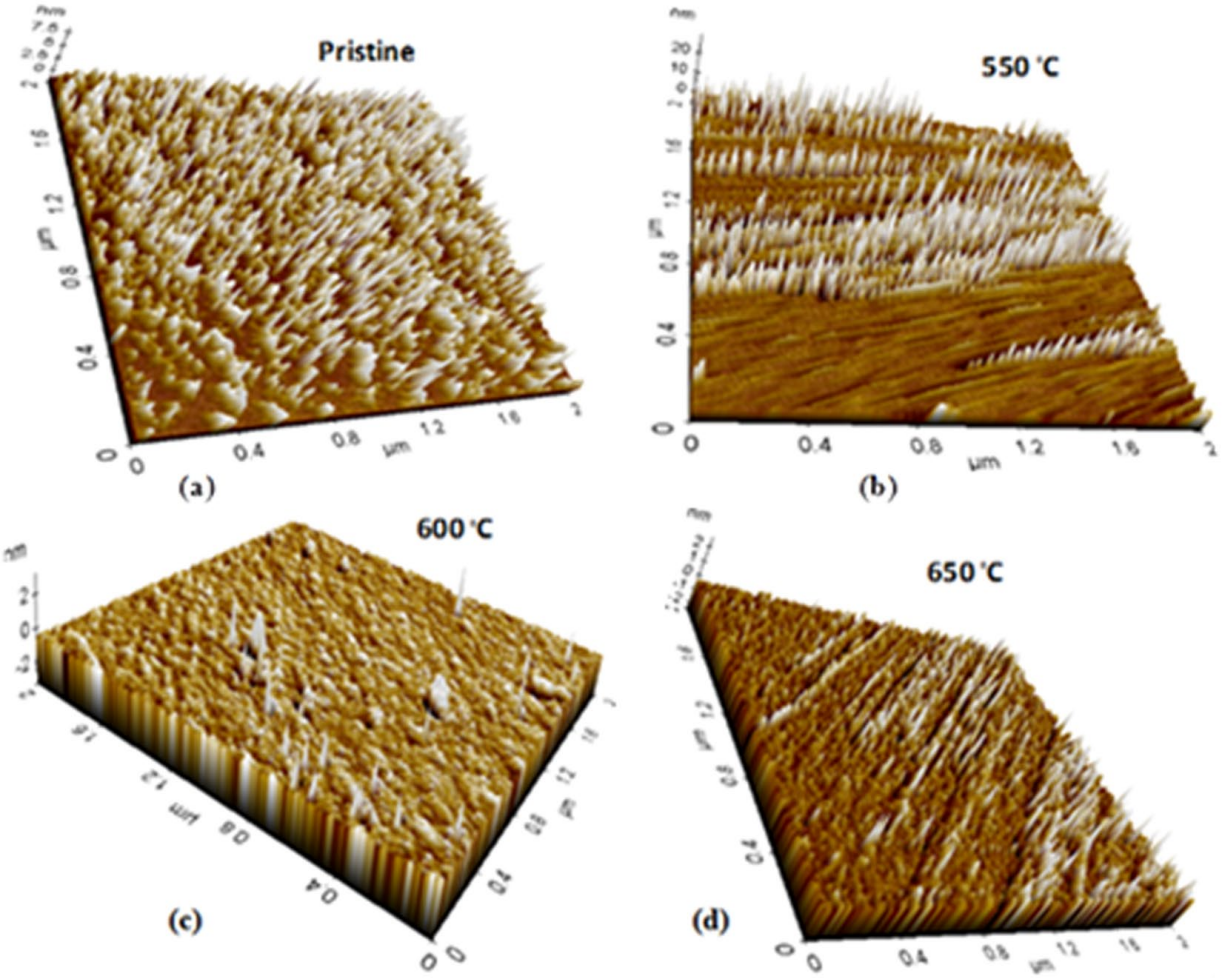
AFM 3D view of pristine and annealed sample, (**a**) pristine (580 ^о^C), (**b**) 550 ^о^C (**c**) 600 and (**d**) 650 ^о^C.

AFM images explain a topographical view of Cu embedded glass materials (Fig. 10). Pristine sample shows the larger numbers of Cu particles on surface due to a presence of Cu ions along with Al, Si, Fe and Mg alkali metals. The glass contents like silicates and other alkali metals show the sharper 3D peaks on surface as the sample is exchanged at 580 °C (Fig. 10a). In the annealed samples, the Cu atoms are occurred on glassy surface because of *in situ* thermal growth from 550 to 650 °C (Fig. 10b–d). The Cu particles are transported towards the relaxed surface on annealing at separate temperatures. The Cu NPs are diffused in variable sizes and reorientation under a higher kinetic energy at higher temperature. The AFM images indicate the pattern and arrangements of Cu NPs on outer surfaces of glass which reflect 3D pattern of Cu atomic orientations^38^. The mean size of Cu NPs increases due to a thermally enhanced diffusion of Cu atoms which contribute to a growth of larger particles. Hence an increase in size after annealing tends to interdiffuse and change a surface morphology of the Cu embedded glass materials.

### Secondary Ion Mass Spectroscopy (SIMS) analysis

**Figure 11:**
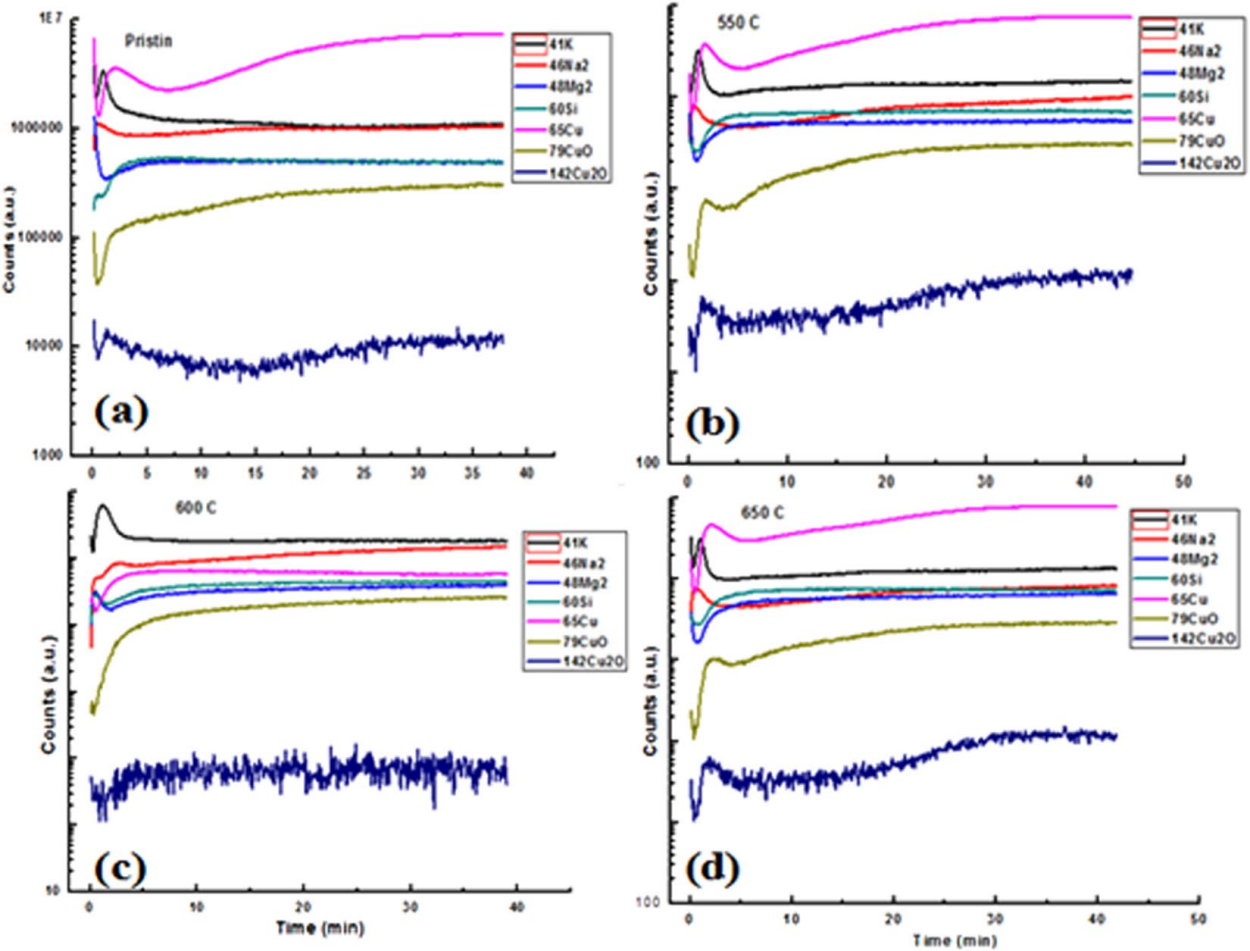
SIMS profiles for copper–alkali ion exchange at variable temperatures. (**a**) Pristine (580 °C), (**b**) 550 °C (**c**) 600 and (**d**) 650 °C.

To investigate embedding nature of Cu NPs, SIMS analysis is performed in which copper and alkali ion concentration profiles are obtained for annealed samples (Fig. 11). We have used 5 keV for O2 as a primary beam at FG-300 emission voltage with a normal-incidence electron gun to compensate the charge that builds up for analysis. The SIMS analysis infers a presence of pure Cu with other alkali metals with a significant difference between the Cu_2_O and Cu^0^ with pure Cu. The spectra of the Cu show the sharply intense peaks as compared to Cu_2_O/Cu^0^ which confirms a higher concentration of pure Cu at higher temperature while the weakly intense peaks indicate a lower concentration of oxide species. The intensified peak of the Cu considered as larger population of neutral atoms over the ionic Cu_2_O/Cu^0^ in the glass matrix (Fig. 11a–d). These results agree with the XPS and the GIXRD results support Cu NPs formation with ascending temperature. Since, the Cu^+^ species are comparably highly polarized and mobile than the Cu^2+^ and both the species respond in SIMS spectra with Cu atoms. The linear spectra for the pure Cu NPs indicate a uniform embedding nature of Cu particles inside the glass matrix from 600 to 650 °C. Both the diffusion and accumulation of Cu depend on dialectic medium and even coordination length of Cu-O with different geometric sites. Hence the Cu-O shows a shorter bond length than the measured Na-O separation inside glass matrix and the Cu atoms follows a Gaussian-like penetration profile for a local rearrangement in glass matrix^18,39^. Thereby the Cu atoms which are diffused at variable size are displaced on higher thermal condition (Fig. 11).

### *In-situ* temperature driven mechanism for Cu NPs

**Figure 12:**
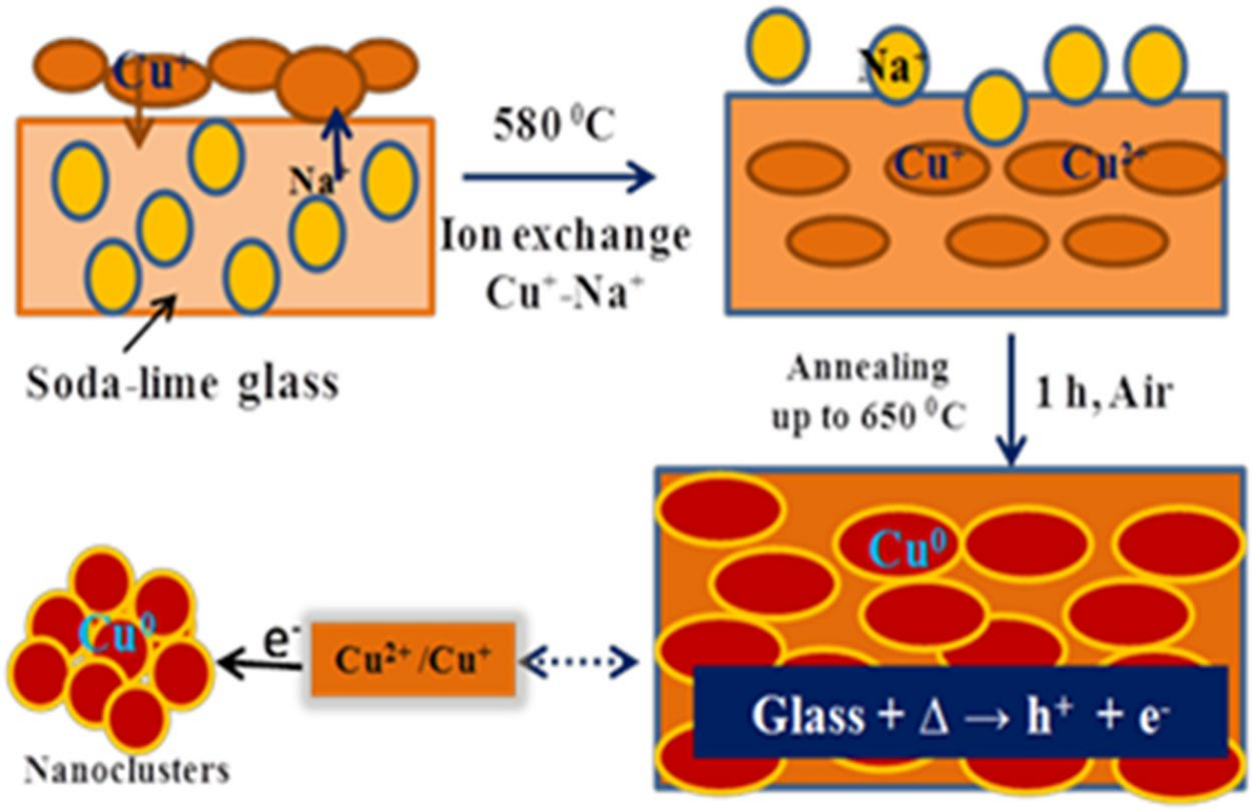
Schematic representation for thermal growth of Cu NPs inside glass matrix.

During an ion exchange process, the Cu^+^/Cu^2+^ ions which are incorporated into host matrix by substituting Na^+^ at 580 °C owing the Cu reduction by capturing required electrons from glass matrix especially from silicate species^40^ (Fig. 12). The population of Cu NPs depends upon a fractional volume of Cu^+^/Cu^2+^ ions which require sufficient electrons for redox reaction in a growth mechanism. In our proposed mechanism the required electrons are captured from glass containing intrinsic oxygen atoms. A thermal reduction of Cu ions into Cu neutral particles is explained by an temperature driven growth mechanism where the available electrons have reduced the Cu ions to Cu metals. The Cu neutral atoms are formed under a higher temperature treatment but the Cu could oxidize during an annealing as the process is performed in an open air atmosphere^37^. In the redox process, the Cu^+^/Cu^2+^ ions are reduced into Cu^0^ by following ionization, reduction and oxidation reactions systematically under a thermal treatment. The Cu atoms with a higher kinetic energy diffuse on glass surfaces at higher temperature because of a thermal relaxation of surface tensile stress as per their surface energy. The Cu^0^ accumulation with a relaxation produces the Cu NPs on glassy surfaces after cooling at RT for 5–6 h. Such spatial Cu NPs arrangement vis-a-vis thermal reduction induces thermodynamic changes. Thus, we have investigated the thermodynamics, ∆H, ∆S and ∆G of metallic nanomaterials under thermal process (Table 2). The temperature driven mechanism for the Cu NPs growth illustrated below in the following equations.45

6 $$\begin{array}{lllll}2{{\rm{O}}}^{-} & \to  & {{\rm{O}}}_{{\rm{2}}}+{{\rm{e}}}^{-} & \quad \to  & {\rm{Oxidation}}\end{array}$$

7 $$\begin{array}{lllll}{{\rm{CuSO}}}_{{\rm{4}}} & \mathop{\longrightarrow }\limits^{{\rm{\Delta }}580^\circ {\rm{C}}} & {{\rm{Cu}}}^{+}{/\mathrm{Cu}}^{2+} & \quad \to  & {\rm{Dissociation}}\end{array}$$

8 $$\begin{array}{lllll}{{\rm{Cu}}}^{+}{/\mathrm{Cu}}^{2+} & \underset{{\rm{\Delta }}650^\circ {\rm{C}}}{\overset{{{\rm{e}}}^{-}}{\longrightarrow }} & {{\rm{Cu}}}^{{\rm{0}}} & \quad \to  & {\rm{Reduction}}\end{array}$$

**Table 2:** The Δ*H*, Δ*S* and Δ*G* values for pristine and annealed samples.

T (k)	1/T	Abs (a. u.)	log (Abs)	*E*a (kJ/m)	H (kJ/m)	G (kJ/m)	S (kJ/m)
923.15	0.00108	0.6	−0.2218	12.5458	−7.6625	3.9213	−0.0125
873.15	0.00114	0.49	−0.3098		−7.2468	5.1794	−0.0142
823.15	0.00121	0.38	−0.4202		−6.8311	6.6230	−0.0163
853.15	0.00117	0.16	−0.7959		−7.0805	13.0010	−0.0235

### Thermodynamic studies for structured Cu NPs in glass matrix

At a low temperature the Cu^+^/Cu^2+^ ions require a sufficient energy to overcome a static potential barrier which could be a bonding energy of the Cu-O following a reduction of Cu^+^/Cu^2+^ to Cu^0^ within the matrix. Thus the required energy is noted as activation energy (*E*_*a*_) which plays a key role for a thermal growth and reorientation of Cu NPs. Thus, we have investigated the activation energy with ∆H, ∆S and ∆G values for both the pristine and annealed processes (Table 2).

**Figure 13:**
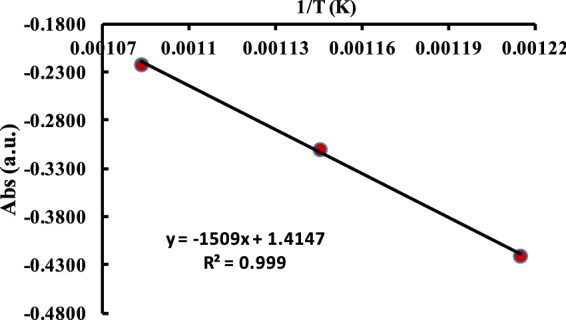
Activation energy of Cu embedded glass sample at variable temperature.

Both the diffusion and accumulation of Cu atoms are increased at a higher temperature which could have caused a change in optical properties by light scattering and absorbance of diffused Cu atoms^41^. Therefore, the UV-Vis absorbance is used to detect changes in form of an optical density at specific wavelength. Thereby, the optical density or absorbance is used as the authentic data to calculate the activation energy along with other thermodynamic properties. Activation energy is calculated using Arrhenius equation on increasing temperature (Supplementary Fig. S2) which is fitted as-

9 $$\mathrm{log}({\rm{abs}})={\rm{logA}}-\frac{{\rm{Ea}}}{2.303{\rm{R}}}\,{\rm{Or}}\,\mathrm{log}({\rm{abs}})={\rm{logA}}-\frac{{\rm{Ea}}}{2.303{\rm{R}}}\frac{1}{{\rm{T}}\,}$$

where, abs = absorbance, T = Temperature (Kelvin), R = Gas constant (8.314 J/mol/k), A = frequency factor, *E*_*a*_ = activation energy (J/mol). The log (abs) vs 1/T plot of Arrhenius equation is a straight line with (−*E*_*a*_/R) slope. It depicts the Cu^0^ diffusion on glass matrix is as a 1^st^ order process (Fig. 13). Reduction from Cu^+^/Cu^2+^ to Cu^0^ with a consequent diffusion of Cu^0^ atoms towards a surface depends on an activation energy which is required for breaking the Cu-O bonds during heating. So the advanced thermodynamic treatment is applied in our study. The Cu^0^ is featured by an optical absorbance and so absorption is noted as an authentic experimental variable for calculating the *E*_*a*_. This *E*_*a*_ calculates the ∆H for pristine and annealed process given as under-

10 $$\mathrm{log}({\rm{abs}})={E}_{a}-nR$$

Temperature effects on pristine and annealed samples are studied, the absorbance vs temperature relation furnishes information about kinetic rate for Cu^0^ clustering. For Cu NPs growth under thermal conditions, the Δ*G* and Δ*S* are calculated using equations (Fig. S2) given as-

11 $${\rm{Entropy}}({\rm{\Delta }}S)=\frac{{\rm{Ea}}-{\rm{RT}}+2.303{\rm{RTlog}}({\rm{abs}})}{{\rm{T}}}$$

Equation is modified as under-

12 $${\rm{\Delta }}S=\frac{[{\rm{Ea}}]}{{\rm{T}}}-{\rm{R}}[1-2.303\,\mathrm{log}({\rm{abs}})$$

Since the UV-Vis absorbance has inferred the activities of absorbance data transformation from Cu^+^/Cu^2+^ → Cu^0^, therefore it is used as a variable in equation for calculating ∆G which is given as–

13 $${\rm{\Delta }}G=-2.303{\rm{RTlog}}({\rm{abs}})$$

where, symbols are usual for Cu NPs growth under an applied thermal treatment at adequate *E*_*a*_ and ∆G both. Thermodynamic parameters infer the thermally induced Cu NPs growth.

**Figure 14:**
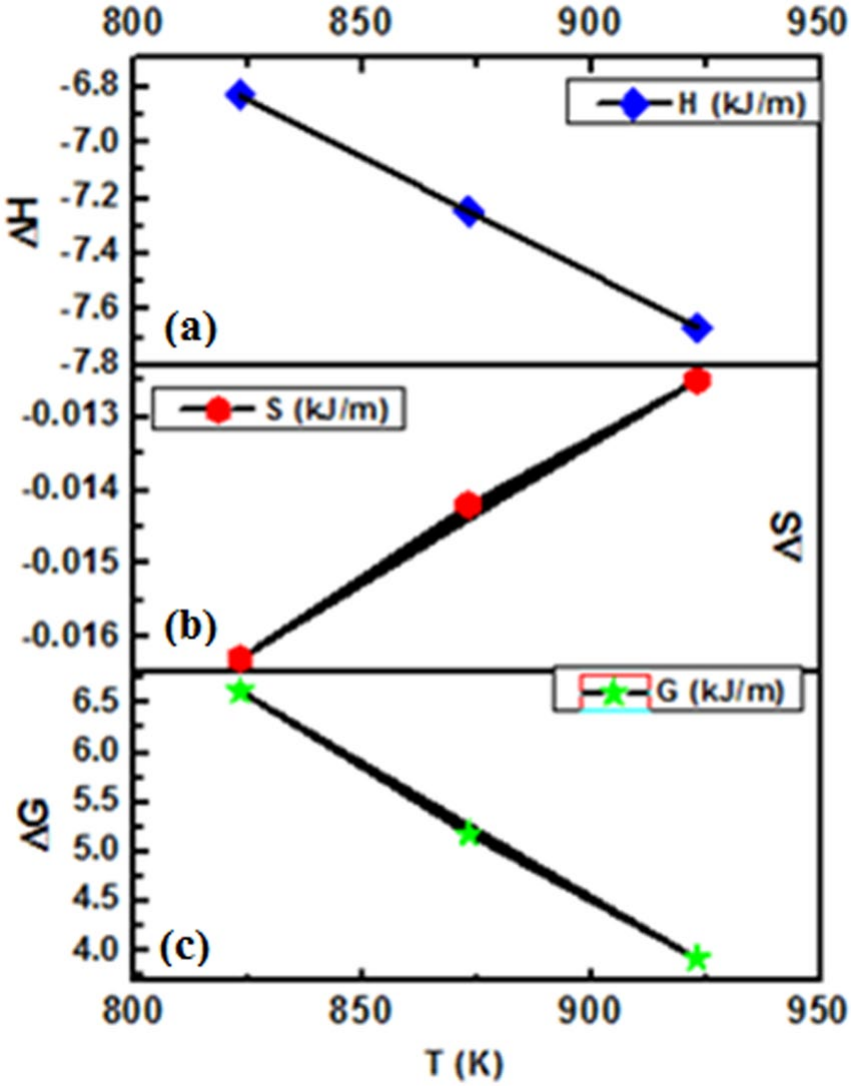
Thermodynamic values (KJ/m) for Cu embedded glass samples. (**a**) ∆H Vs T, (**b**) ∆S Vs T and (**c**) ∆G Vs T (K).

The Δ*H* from −6.8311to −7.6625 KJ/mol depicts Cu growth and nucleation processes for annealed samples. Δ*H* from 823.15 to 923.15 K becomes a endothermic may be due to the higher energy involvment for bond breaking phenomenon of Cu^0^/Cu_2_O species inside the glass matrix. The Δ*S* values from −0.0235 to −0.0125 kJ/m depict a favourable entropic change with increased Cu^0^ diffused and its reorientation in the glass matrix. Both, the Δ*H* and Δ*S* values show a most optimized energy with a mutual relationship of enthalpy-entropy compensation phenomenon with an inverse trend of their values (Fig. 14a,b). Such trends depict causing either the higher Δ*H* absorption or a release and in both the cases, the Δ*S* increases and decreases accordingly. The ∆G values from 13.0010 to 3.9213 923.15 for 853.15 to 923.15 K indicate a spontaneous process for the reduction and diffusion of Cu atoms (Table 2). On increasing positive Δ*H* values with similar decrease in ΔG values reflect a coercive thermodynamic energy exchange from 580–650 °C (Fig. 14a,c). Thereby the thermodynamic relationship elucidates a need of kinetic energy required for transforming Cu^+^/Cu^2+^ to Cu^0^ and accumulating the Cu atoms towards glassy surface for developing optimized arrays^42,43^. Thus their mutual relationship depicts a partitioning of Cu NPs homogeneous distribution in glass matrix.

### Thermodynamic synergy among Δ*G*, Δ*H* and Δ*S*

**Figure 15:**
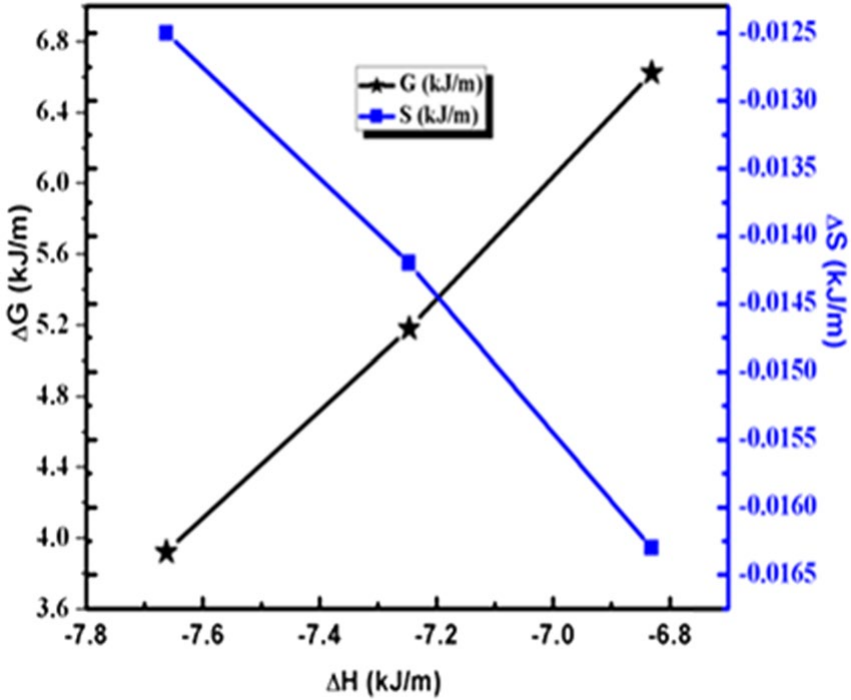
Kinetic model for enthalpy, entropy and Gibbs energy for pristine and annealed Cu embedded glass samples.

A mutual relation among Δ*G*, Δ*H* and Δ*S* reveals thermal growth of Cu atoms with respect to temperatures in glass matrix. The Δ*S* and Δ*H* both have shown a reverse trend with respect to Δ*G*. On increasing the Δ*H*, the Δ*S* is increased due to a required thermal energy transformation during the bond breaking and making processes of Cu-O and Cu-Cu atoms, respectively. The ∆H, ∆S and ∆G mutually explain the stability and effective distribution of Cu NPs with their spontaneity behaviour (Fig. 15). The ∆H values revealed the reduction process of reduced Cu atoms through a redox while ∆S helps to understand the Cu NPs reorientation mechanism which was reported in our previous work^44^. These two thermodynamic parameters collectively have inferred thermal growth and accumulation behaviour which utilize almost all the energy in a process to balance a minimum energy. Spontaneity of any chemical reaction could be represented by Δ*G* values^45^ and thus, we have explained a dependence of Δ*G* in favour of Cu NPs formation within dielectric matrix. During the growth and nucleation of Cu atoms, a decrease in Δ*G* value is observed with an increase in annealing temperatures. This may be materialized due to other thermodynamic parameters which followed reverse manner with Δ*G* and at a higher temperature the Cu atoms tend to self-accumulate with a higher kinetic energy. The critical point of thermodynamic synergy is that the Δ*G*, Δ*H* and Δ*S* data are dependent and interdependent to each other as per chemical process for Cu reduction and growth. In our studies, trend and magnitude of ΔH, ΔS and ΔG model have been developed for nucleation and growth of the Cu^0^ under a thermal process from 550–650 °C.

## Conclusion

The Cu NPs were grown and nucleated in a glass matrix by an ion exchange process followed by a thermal annealing from 550–650 °C. The Cu ions are reduced into neutral copper atoms (Cu^0^) and subsequently the Cu^0^ atoms were diffused towards the surface under thermal treatments. The SPR behavior with different sizes of the Cu NPs is determined and explained using UV-Vis measurements at various temperatures. Raman and SIMS spectra have shown clear evidence in favour of Cu NPs growth and diffusion on glass matrix interface under temperature induced process. HRTEM images have confirmed the spherical Cu NPs (d_111_ = 0.20 nm) with 16.5 nm average particle size at 650 °C which is consistent with Mie theory based results. GIXRD result has revealed that the pure and oxide phase of Cu NPs at various temperatures similar to the XPS results. XPS spectrum has confirmed the oxidation state of Cu^0^ atoms which is shown a presence of Cu element in the glass matrix at 650 °C. Thermodynamic studies are explained for a thermally induced growth mechanism of Cu NPs inside glass matrix where activation energy of 12.54 KJ/mol is calculated. The entropy, enthalpy and Gibbs energy for the thermal annealed samples were studied which give a systematic information for nucleation and growth of Cu^0^ neutral atoms w.r.t. annealed temperatures. The thermodynamic synergy explained the growth and reorientation of Cu NPs inside the glass matrix. This fundamental study could be significant to understand a temperature driven growth mechanism with optical response for embedded metallic nanostructures for optoelectronic uses.

## Experimental Section

### Chemicals and materials

Chemical reagents CuSO_4_ (99.0%) and NaSO_4_ (99.0%) metal salts were procured from sigma Aldrich. Commercial soda-lime glass with weight % of 72.0% SiO_2_, 14.0% Na_2_O, 0.6% K_2_O, 7.1% CaO, 4.0% MgO, 1.9% Al_2_O_3_, 0.1% Fe_2_O_3_, and 0.3% SO_3_) compositions with 1 mm thickness (Blue Star Company, India) were used as a host matrix.

### Temperature driven Cu^+^/Cu^2+^ ion exchanged process

**Figure 16:**
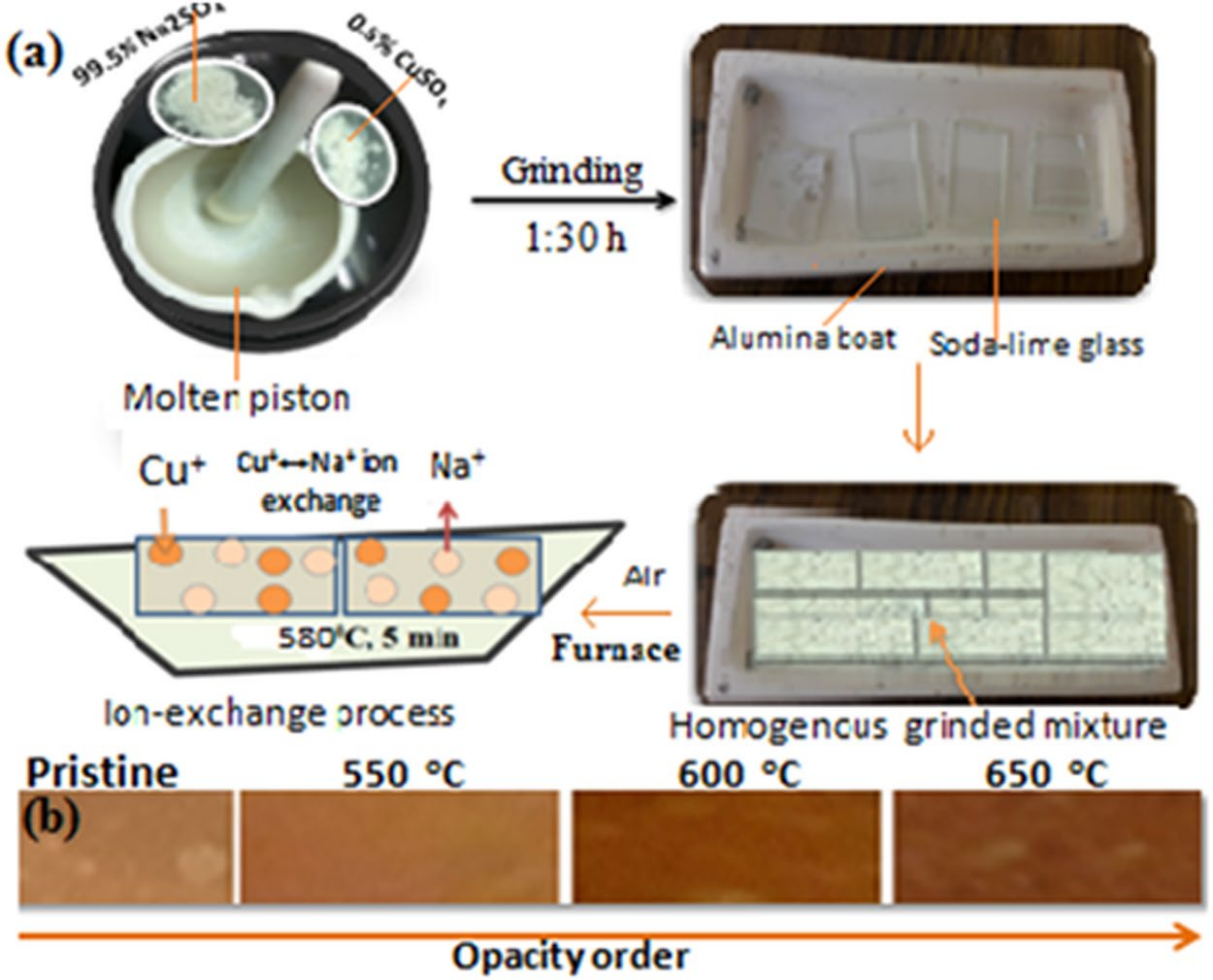
Ion-exchange process for embedding Cu NPs inside glass matrix. (**a**) An ion-exchange process followed by thermal annealing upto 650 °C. (**b**) Optical response for the pristine and annealed samples.

The Cu embedded glass material was prepared by an ion exchange method on thermal annealing from 550–650 °C in an air atmosphere for 1 h. For Cu embedding, the soda-lime glass was chosen as a dielectric host matrix for Cu NPs growth. Initially, the glass slides were poured in formic acid for 15 min for removing impurities from slide surfaces. Later, poured glass slides were cleaned with distilled water, acetone and trichloroethylene by ultrasonication (20 KHz) for 15 min for ultra pure surfaces. Now, the 0.5% CuSO_4_ and 95.5% NaSO_4_ homogeneous mixture was prepared by molten piston grinding. The glass slide pieces were kept into Alumina boat (Al_2_O_3_ > 99%) and filled with grinded CuSO_4_ and Na_2_SO_4_ homogeneous mixture. Further, the alumina boat was transferred into the tubular furnace for the Cu and Na ion exchange inside a glass slide at 580 °C for 5 min. During 5 min duration, the Cu^+^ ions diffused into glass matrix to replace Na^+^ ions (Fig. 16a). After ionic *in situ* diffusion as ion-exchanged samples were cooled at RT followed by cleaning with distilled water and acetone for removing unused CuSO_4_ from glass surface. Ion-exchange process followed by annealing upto 650 °C.

Ion-exchanged (pristine) samples initially were faint yellow or colourless and this optical behaviour observed due to the *in situ* Cu^+^/Na^+^ diffusion with different sizes, mechanical stress and change in electrical polarization of diffused ions in glass (Fig. 16b). Further, the cleaned pristine samples were annealed from 550 °C to 650 °C for 1 h. At lower temperature, the few Cu ions were formed by Cu-O bond breaking and the ions required energy for moving towards a more relaxed surface. Thus, on increasing temperature larger Cu segregation and diffusion were occurred on glassy surfaces which was understood by the differences in the sizes of Cu^+^ and Na^+^^44,46,47^.

### Characterization of Cu^0^ embedded glass materials

Growth of Cu^0^ inside glass matrix was optimized by thermal annealing from 550 to 650 °C using tubular furnace. Plasmonic properties were studied by dual beam UV-Visible spectroscopy (spectro 2060 plus) under a range of 200–800 nm wavelength and pure soda-lime glass was used as a blank sample for UV-Vis measurements. The PL measurements were carried out by Fluoromax photoluminescence spectrometer at 325 nm exciting wavelength for all the samples. The shape and size of Cu^0^ NPs were studied with HRTEM (JEOL TEM 2100) operated at 200 kV. For sample preparation, the Cu embedded glass sample was crushed via mortar and pestle for forming a fine powder and then few milligrams of the powder were dispersed in ethanol on ultrasonication for 30 min. Few drops of upper suspension was placed on carbon copper coated grid with 200-mesh for analysis. Near field Scanning Optical Microscope with Raman spectrometer, Witec, Germany was done using for both the pristine and annealed samples. GIXRD (3 kW X-ray generator with Cu and Mo targets) was used to confirm a crystallinity of Cu NPs with their oxide phases in glass matrix. XPS measurements were performed using an Omicron Nanotechnology ESCA plus (electron spectroscopy for chemical analysis) with UHV twin anode Al K_α_ radiation 1486.6 eV, generated by 15 kV electron effect on Al anode. 20 eV pass energy was applied for 0.5 eV resolutions during photoelectron scanning. SIMS technique (HIDEN ANALYTICAL) was used for the deft profile of embedded nanoparticles inside glass matrix by applying 5 KeV energy at FG-300 emission voltage.

## Electronic supplementary material

Supplementary Information (SI)
