# Supplementary material for: Thermodynamically induced in Situ and Tunable Cu Plasmonic Behaviour
Source: Sci Rep. 2018 Feb 14;8:3006. doi: 10.1038/s41598-018-20478-y (PMC5813046; doi:10.1038/s41598-018-20478-y)
Supplement: Supplementary file 1 — Supplementary Information (SI) [file 41598_2018_20478_MOESM1_ESM.pdf]

Supplementary Information

Thermodynamically induced *in Situ* and Tunable Cu Plasmonic Behaviour

Gajendra Kumar Inwati[1], Yashvant Rao[2] and Man Singh[3*]
[1]Centre for Nanosciences, Central University of Gujarat, Gandhainagar-382030, India.

[2]Centre for Nanosciences, Central University of Gujarat, Gandhainagar-382030, India.

[3]School of Chemical Sciences, Central University of Gujarat, Gandhainagar-382030, India.

[*]mansingh50@hotmail.com

Experiment details

Material. The $CuSO_4$ (99.0%) metal salt and, $NaNO_3$ (99.0%) were purchased from sigma Aldrich. Commercial soda-lime glass with composition (weight %) of 72.0% SiO_2, 14.0% Na_2O, 0.6% K_2O, 7.1% CaO, 4.0% MgO, 1.9% Al_2O_3, 0.1% Fe_2O_3, and 0.3% SO_3) and thickness of 1 mm were used. Ion exchange rout has been performed for the synthesis of Ag embedded soda lime-glass.

Method. Soda-lime glasses were poured in formic acid for 15 min to remove the impurity from surface. The poured slides were cleaned with distilled water, acetone and trichloroethylene by ultrasonication (20 KHz) for 15 min. The homogeneous mixture of 0.5% $CuSO_4$ and 95.5% $NaNO_3$ were prepared by molten piston grinding. The glass slide pieces were kept into the Alumina boat (Al_2O_3> 99%) and filled with grinded homogeneous mixture of precursors. The alumina boat transferred into the tubular furnace for Cu and Na ion exchange inside the glass slide at 390 °C for 5 min at air atmosphere. The as ion-exchanged samples were cooled at room temperature and cleaned with distilled water and acetone. After cleaning the samples were annealed from 500° C to 650 °C for 1 h so that the glass melting point can be maintained to prevent soda-lime glass. The annealed samples were taken for the investigation of structural and optical behaviour of the samples.

1.1 Ion exchange method

In ion exchange method, the immersed glass with molten Cu and Na ions diffuse from the molten salts to the glass surface under thermal treatment. Cu ions embedded in glass matrix from the Cu molten salt by replacing sodium ions (Fig. 1). The embedded Cu ions change the compositions of silicate glass by replacing the sodium ions are given in temperature driven mechanism in the manuscript.

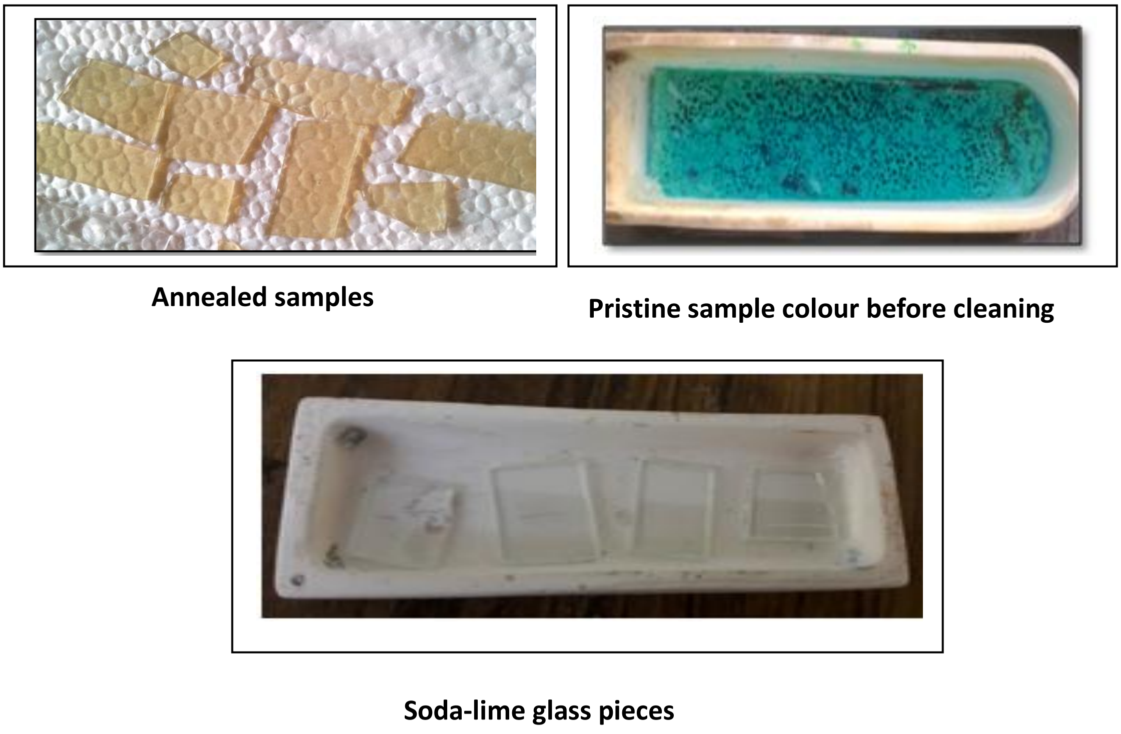

Figure S 1. Colourization of pristine and annealed cu embedded samples

3. Equations S2

The Arrhenius equation is given as-

$$k = Ae^{-E_a/(RT)} \qquad (1)$$

Modified equation given as-

$$\log(abs) = \log A - \frac{E_a}{2.303R} \qquad (2)$$

Thermodynamic parameters are expressed as-

$$\Delta S = \Delta H - \Delta G / T \qquad (3)$$

Figure S2. Arhenius and thermodynamic equations for ΔH, ΔS, ΔG and E_a calculation

3. Raman microscopic images

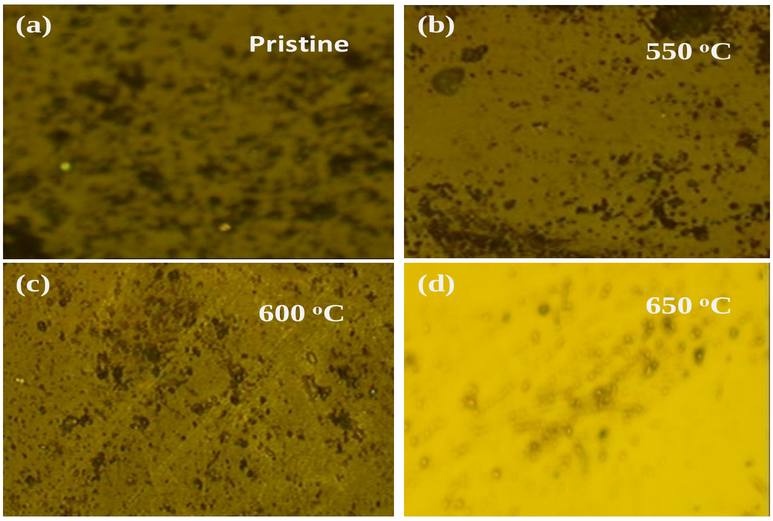

Figure S3. Raman micro-images of Cu embedded glass samples

4. Crystal structures of Cu atoms

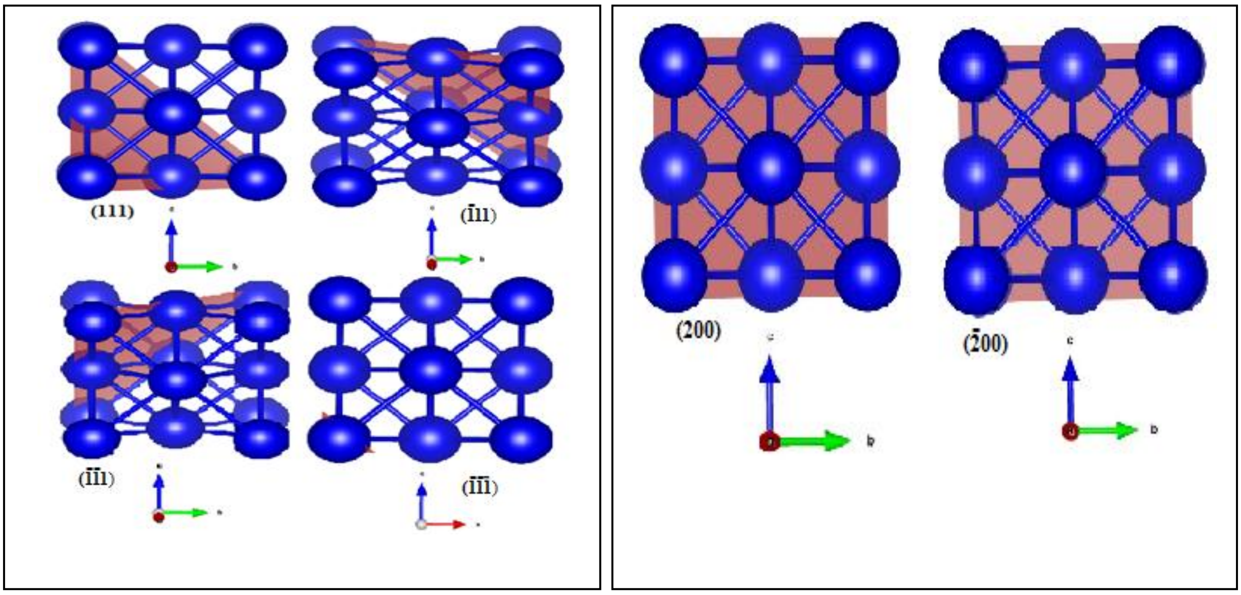

Figure S4. Crystallographic planes of Cu crystal structures
